# Elevated rates and biased spectra of mutations in anaerobically cultured lactic acid bacteria

**DOI:** 10.1128/mbio.03054-25

**Published:** 2025-11-13

**Authors:** Owen F. Hale, Michelle Yin, Megan G. Behringer

**Affiliations:** 1 Department of Biological Sciences, Vanderbilt University 5718, Nashville, Tennessee, USA; 2 Evolutionary Studies Initiative, Vanderbilt University 5718, Nashville, Tennessee, USA; 3 School of Medicine, Tulane University 5783, New Orleans, Louisiana, USA; 4 Department of Pathology, Microbiology, and Immunology, Vanderbilt University Medical Center 12328, Nashville, Tennessee, USA; California State University Northridge, Northridge, California, USA

**Keywords:** neutral evolution, mutation rate, spontaneous deamination, *Lactobacillus*, CRISPR

## Abstract

The rate, spectrum, and biases of mutations represent a fundamental force shaping biological evolution. Convention often attributes oxidative DNA damage as a major driver of spontaneous mutations. Yet, despite the contribution of oxygen to mutagenesis and the ecological, industrial, and biomedical importance of anaerobic organisms, relatively little is known about the mutation rates and spectra of anaerobic species. Here, we present the rates and spectra of spontaneous mutations assessed anaerobically over 1,000 generations for three fermentative lactic acid bacteria species with varying levels of aerotolerance: *Lactobacillus acidophilus*, *Lactobacillus crispatus*, and *Lactococcus lactis*. Our findings reveal highly elevated mutation rates compared to the average rates observed in aerobically respiring bacteria, with mutations strongly biased toward transitions, emphasizing the prevalence of spontaneous deamination in these anaerobic species and highlighting the inherent fragility of purines even under conditions that minimize oxidative stress. Beyond these overarching patterns, we identify several novel mutation dynamics: positional mutation bias around the origin of replication in *Lb. acidophilus*; a significant disparity between observed and equilibrium GC content in *Lc. lactis*; and repeated independent deletions of spacer sequences from within the clustered, regularly interspaced, short palindromic repeat locus in *Lb. crispatus,* providing mechanistic insights into the evolution of bacterial adaptive immunity. Overall, our study provides new insights into the mutational landscape of anaerobes, revealing how non-oxygenic factors shape mutation rates and influence genome evolution.

**IMPORTANCE:**

Despite Earth’s oxygen-free origins and the abundance of microorganisms that thrive without oxygen, little is known about the rates and patterns of mutations in anaerobic species. This study directly measures the mutation rates of three fermentative lactic acid bacteria across 1,000 generations in oxygen-free conditions, revealing elevated and highly biased genome-wide mutation rates in these species compared to oxygen-using bacteria. These results highlight how non-oxygen-related factors contribute to mutagenesis, shaping genome evolution in microbes with anaerobic life histories.

## INTRODUCTION

Life on Earth began in an anaerobic environment that remained so for approximately 2 billion years (1). With the advent of oxygenic photosynthesis, many organisms adapted to harness the redox potential of molecular oxygen to more efficiently generate energy from available nutrients (2, 3). However, aerobic respiration evolved on the preexisting chassis of anaerobic respiration, leaving anaerobes vulnerable to oxidative stress caused by, and targeted to, the highly reactive enzymes needed for efficient anaerobic metabolism (4, 5). In response, some species evolved behaviors and/or complex machinery for protecting their cells from oxidative damage, while others remodeled their physiology to tolerate transient exposure to oxygen (5). In addition to the historical importance of anaerobes and the transition to an aerobic atmosphere, anaerobes today represent important environmental (6, 7), industrial (8), and pathogenic (9) members of the biosphere, and the role of oxygen in driving evolution through mutagenesis has been well characterized (10, 11). Despite this, little is known about the mutation rates and spectra of anaerobic organisms.

Characterizing the rate at which mutations occur and the relative rates of specific subtypes of mutations, also known as the mutation spectrum, is critical for biological research, as mutation provides the raw material of genetic diversity on which the other forces of evolution can act, is directly influenced by a species’ evolutionary past, and constrains a species’ evolutionary future. To date, mutational studies have been completed for over 150 organisms across the tree of life and scale of organismal complexity, providing a deep understanding of mutation rates and highlighting the role of oxidative damage in shaping the mutation rate and spectrum (11–13). However, despite the curation of such a rich data set of mutational patterns, quantification of mutation rate and spectrum has primarily focused on species with aerobic metabolisms under oxygenated conditions. Because of the requirement for specialized equipment and the fastidious and/or pathogenic nature of many anaerobes, mutation rates have only been directly quantified for two anaerobically cultured organisms to date (14, 15). As anaerobes include important environmental, industrial, and pathogenic taxa and occupy a wide range of phylogenetic and ecological diversity, there exists a need to thoroughly investigate the causes and consequences of mutation across the anaerobic tree of life.

**Fig 1:**
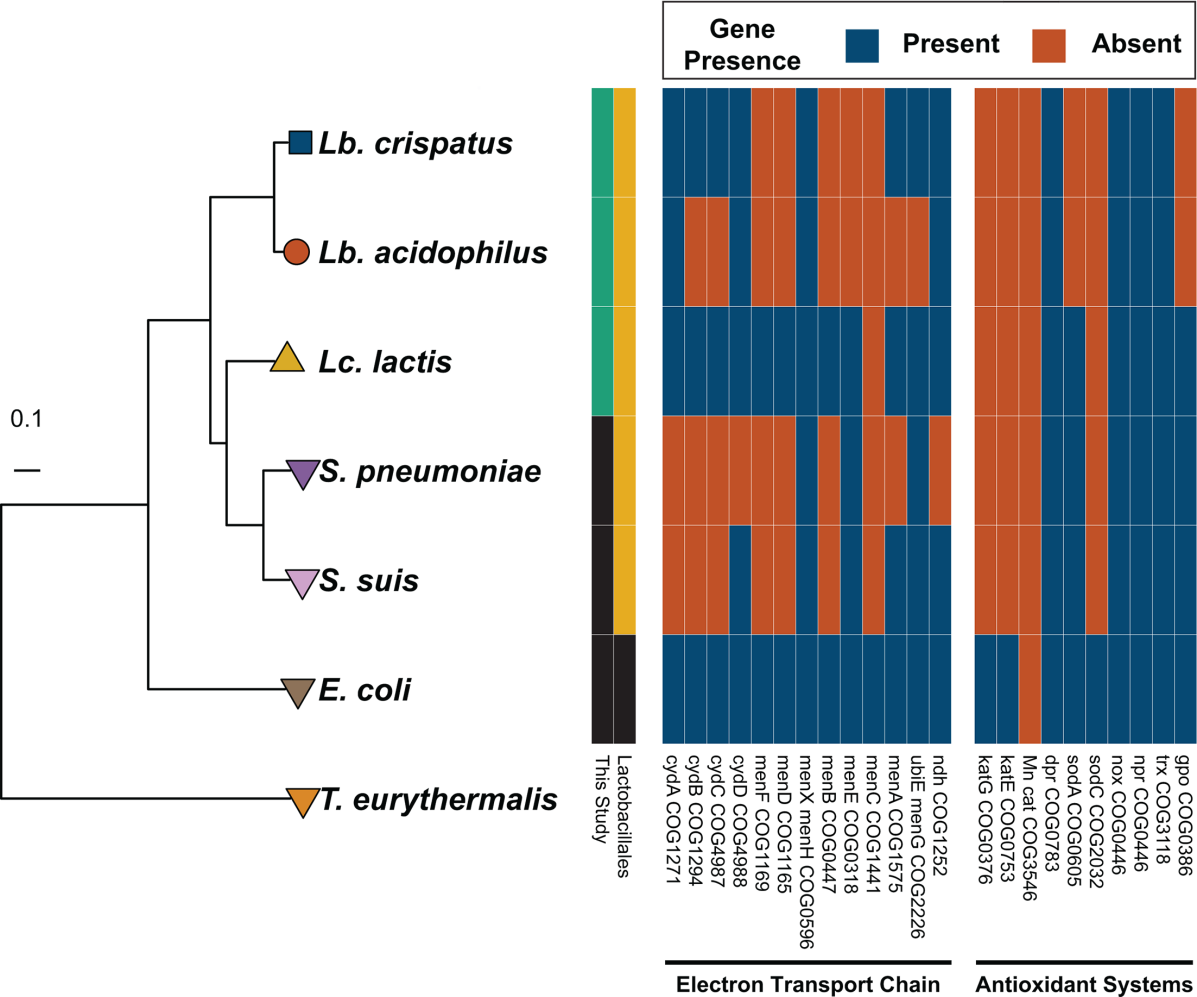
Phylogenetic relatedness and oxidative stress-related gene carriage of notable prokaryotes with mutation rates measured via mutation accumulation experiments coupled with whole-genome sequencing (MA/WGS). Maximum-likelihood phylogeny of prokaryotes with mutations measured in this study (*Lactobacillus acidophilus*, *Lactobacillus crispatus*, and *Lactococcus lactis*) and previous (*Escherichia coli*, *Streptococcus pneumoniae*, *Streptococcus suis*, and *Thermococcus eurythermalis*) MA/WGS experiments, constructed from the *rpoB* gene. The scale bar represents substitutions per site. The left heatmap indicates the species used in this study (green) as well as species in the order Lactobacillales (yellow). The other two heatmaps indicate the presence (blue) and absence (red) of genes involved in the electron transport chain (middle) and antioxidant systems (right). *T. eurythermalis* was excluded from the heatmap due to its evolutionary divergence from bacteria. cydA, cytochrome *bd*-I ubiquinol oxidase subunit 1; cydB, cytochrome *bd*-I ubiquinol oxidase subunit 2; cydC, ATP-binding cassette transporter CydC; cydD, ATP-binding cassette transporter CydD; menF, menaquinone-specific isochorismate synthase; menD, 2-succinyl-5-enolpyruvyl-6- cyclohexadiene-1-carboxylate synthase; menX, 2-succinyl-6-hydroxy-2,4-hydroxy-3-cyclohexene-1-carboxylate synthase; menB, naphthoate synthase; menE, o-succinylbenzoic acid-CoA ligase; menC, o-succinylbenzoate synthase; menA, 1,4-dihydroxy-2-naphthoate octaprenyltransferase; ubiE, demethylmenaquinone methyltransferase; ndh, NADH dehydrogenase; katG, catalase-peroxidase; katE, catalase; Mn cat, manganese-dependent catalase; dpr, Dps-like peroxide resistance protein; sodA, Mn superoxide dismutase; sodC, Cu-Zn superoxide dismutase; nox, NADH-dependent oxidase; npr, NADH-dependent peroxidase; trx, thioredoxin; gpo, glutathione peroxidase.

Lactic acid bacteria (LAB) are a well-suited group of organisms with which to begin this work. LAB are a polyphyletic group of bacteria known for their production of lactic acid and include, among others, members of the order Lactobacillales (16). Most LAB are classified as aerotolerant or facultative anaerobes with fermentative lifestyles and can be found in diverse habitats, from plants to insects to mammals (17, 18). Importantly, many have been linked to beneficial health effects in humans (19–22) and are used in industrial processes that span dairy fermentation (23, 24) and the production of recombinant therapeutics (23). The Lactobacillales are generally incapable of performing aerobic respiration, and those that can often cannot construct a complete electron transport chain without exogenous heme (25, 26). These organisms also vary in their susceptibility to oxidative stress and possess few genes encoding enzymes that mitigate oxidative damage, such as catalase (Fig. 1). Previous investigations of spontaneous mutations in LAB revealed mutation spectra biased toward mutations at G:C base pairs—positions uniquely susceptible to oxidative damage—which aligns with expectations given these organisms’ propensity for producing DNA-damaging radicals (13, 27–29). The diversity of these organisms, both in their lifestyles and physiologies, as well as their low frequency of pathogenicity and ease of culture, makes them an ideal system for studying the evolution of the mutation rate and spectrum.

Mutation accumulation experiments coupled with whole-genome sequencing (MA/WGS), the gold-standard method for measuring an organism’s mutation rate and spectrum, have been performed on three aerobically cultured LAB: *Lactococcus lactis* (29), *Streptococcus pneumoniae* (13), and *Streptococcus suis* (30). In all three instances, high mutation rates and biased mutation spectra were observed, although it is unclear whether these observations are the result of susceptibility to oxidative DNA damage rather than an intrinsically higher mutation rate. In MA/WGS experiments, replicate populations of isogenic microbial strains are continuously cultured through single-cell bottlenecks, whole-genome sequenced, and compared to the experiment’s ancestral strain to identify new mutations (31, 32). This allows for the accumulation of spontaneous mutations and minimizes the influence of selection on the observed spectrum of mutations. MA/WGS confers advantages relative to other methods, such as fluctuation tests, because it reliably and repeatably measures the mutation rate and spectrum across the entire genome, enabling the observation of genome-wide mutational patterns (33–37). The wealth of data generated by MA/WGS experiments has enabled evolutionary theorists to test hypotheses about the determinants of the mutation rate. The current leading hypothesis concerning the evolutionary factors shaping mutation rates is the drift-barrier hypothesis, which predicts that selection will act to minimize mutation rates, with the power of genetic drift limiting the efficacy of selection in small populations (38). Given that many LAB are adapted to living within a host (39), a lifestyle associated with a decreased effective population size (N_e_) and therefore increased drift (40), we expect host-adapted LAB to have high mutation rates relative to free-living bacteria.

Here, we performed MA/WGS experiments on three anaerobically cultured members of the order Lactobacillales: *Lactobacillus acidophilus*, a vertebrate gut commensal (41, 42) used in dairy fermentations (41, 43); *Lactobacillus crispatus*, a commensal of the human gut (44, 45) and urogenital tract (22, 44–46) as well as the guts of poultry (44, 47) and sometimes found in sourdough starter (48); and *Lactococcus lactis* subsp. *lactis*, a human gut commensal used in dairy and other industrial fermentation processes (18, 29). For all three species, we calculated the per-base-pair substitution rate, per-base-pair insertion and deletion (indel) rate, and genome-wide mutational biases, and characterized the mutation spectra and occurrence of structural variants (SVs). We compare these results to aerobically cultured *Escherichia coli* (49), three previously studied but aerobically cultured Lactobacillales (13, 29, 30), and the anaerobically cultured hyperthermophilic archaeon, *Thermococcus eurythermalis* (15). To our knowledge, this work represents the most extensive characterization of anaerobic mutation rates to date, markedly increasing our understanding of how the presence or absence of oxygen influences the evolutionary and mutational landscape.

## RESULTS

### Lactic acid bacteria exhibit extremely high single-nucleotide mutation rates

**Fig 2:**
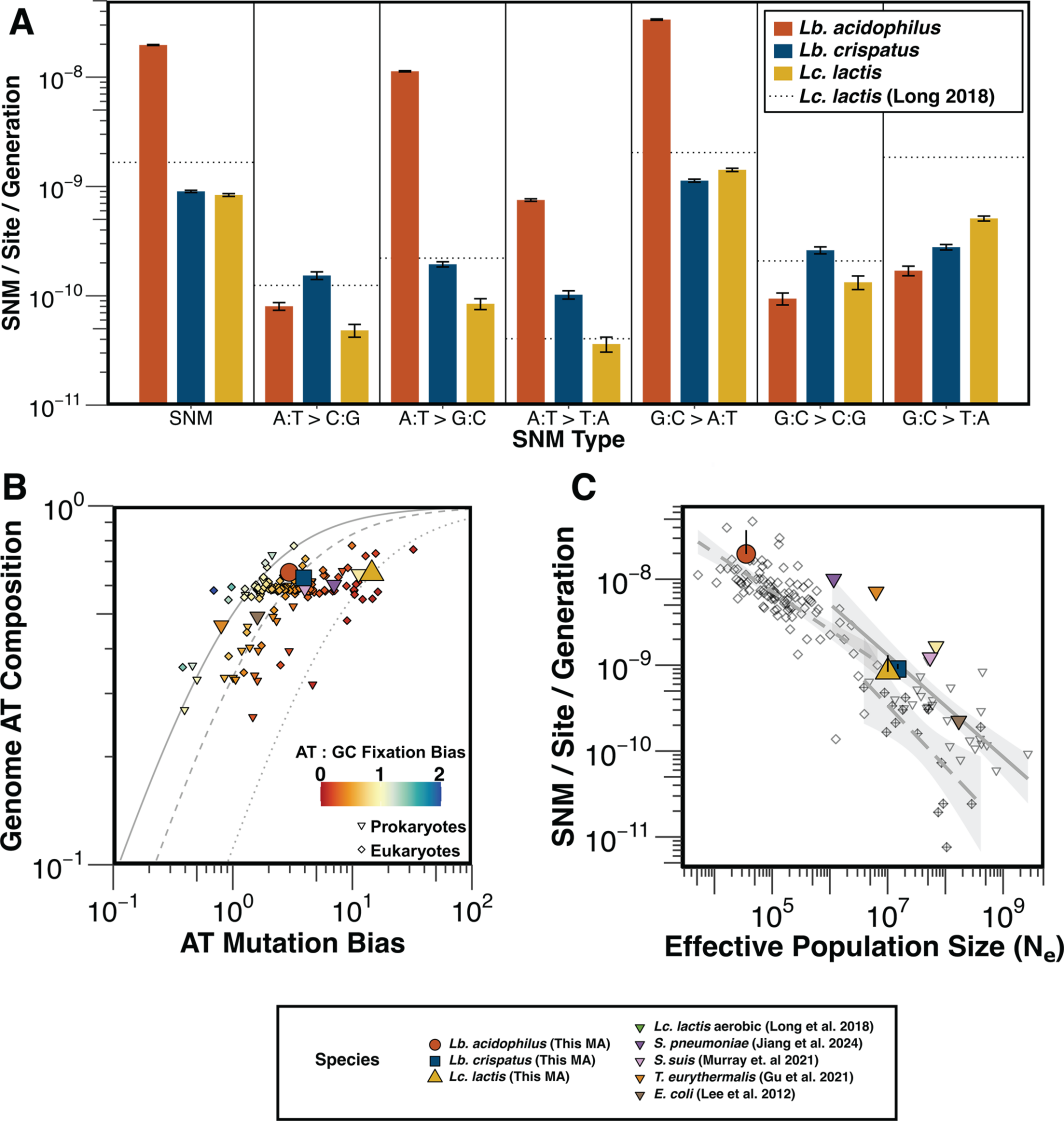
High rates and biased spectra of SNMs in anaerobically cultured LAB. (**A**) Per-site per-generation rates of all SNMs and each of the six SNM types. The dotted black lines represent the previously measured mutation rate and spectrum of aerobically cultured *Lc. lactis* (29). Bars show rate means, and error bars represent 95% confidence intervals. (**B**) Relationship between AT mutation bias, AT composition, and AT fixation bias in diverse organisms. Reference lines for AT:GC fixation biases of 1:1 (solid), 1:2 (dashed), and 1:8 (dotted) are included. Taxa of interest are colored according to the key at the bottom of the figure, while the rest are colored by AT:GC fixation bias according to the key inside the panel. (**C**) Relationship between N_e_ and mutation rate. Organisms are split into prokaryotes (solid line and inverted triangles), unicellular eukaryotes (long dashed line and crossed diamonds), and multicellular eukaryotes (short dashed line, empty diamonds). For *Lb. acidophilus*, *Lb. crispatus*, and *Lc. lactis*, the difference between the observed mutation rate and the mutation rate predicted by the linear regression model created with previously reported prokaryotic mutation rates and N_e_ values is represented by a black line. For a description of the external data used in panels B and C, see the Materials and Methods.

Mutation accumulation for 66 lines of *Lb. acidophilus*, 66 lines of *Lb. crispatus*, and 50 lines of *Lc. lactis* was conducted over 1,171, 1,084, and 1,067 generations, respectively. Across these lines, we observed a total of 3,009 single-nucleotide mutations (SNMs) in *Lb. acidophilus*, 140 in *Lb. crispatus*, and 107 in *Lc. lactis*, for a genome-wide per-base-pair per-generation substitution rate of 1.97 × 10^−8^ (standard error [SE]: 9.79 × 10^−11^), 9.01 × 10^−10^ (SE: 1.11 × 10^−11^), and 8.36 × 10^−10^ (SE: 1.24 × 10^−11^), respectively (Table 1; Fig. 2A). The SNM rate we observe in *Lb. acidophilus* is, to our knowledge, the highest rate recorded in a MA/WGS experiment on a bacterium that has not been genetically engineered. However, it is just below that of *Mycoplasma mycoides* strains JCVI-syn1.0 (mean: 3.13 × 10^−8^, SE: 0.12 × 10^−8^) and JCVI-syn3B (mean: 3.25 × 10^−8^, SE: 0.16 × 10^−8^), two heavily engineered laboratory strains reported to have the highest recorded mutation rates of any cellular organisms (50). These rates are considerably higher than previously examined bacterial SNM rates, such as the *E. coli* K-12 rate (49) of 2.2 × 10^−10^, but similar to the SNM rates of *Lc. lactis* (1.66 × 10^−9^) (29), *S. pneumoniae* (1.01 × 10^−8^) (13), and *S. suis* (1.23 × 10^−9^) (30) in aerobic conditions, suggesting that these higher mutation rates may be a general feature of LAB (Fig. 2C). In fact, coupling our results with a data set of 39 recorded mutation rates from non-engineered bacterial strains reveals that LAB make up 5 of the top 8 bacterial species with the highest mutation rates (12).

**TABLE 1:** Summary of mutation accumulation results

Species	Chr. size	GC content (%)	Fourfold synonymous π per site	Effective population size	# of MA lines	# of Transfers	SNMs			Complex/ MNM	Small insertions			Small deletions		
							Total	Rate^*a*^	SE	Total	Total	Rate^*a*^	SE	Total	Rate^*a*^	SE
*Lb. acidophilus*	1,978,850	34.7	1.40E − 03	3.55E + 04	66	50	3,009	1.97E − 08	9.79e − 11	53	359	2.35E − 09	1.81E − 11	683	4.47E − 09	2.18E − 11
*Lb. crispatus*	2,239,089	37	2.70E − 02	1.50E + 07	64	50	140	9.01E − 10	1.11E − 11	13	18	1.16E − 10	3.14E − 12	53	3.41E − 10	6.02E − 12
*Lc. lactis*	2,399,762	35.2	1.69E − 02	1.01E + 07	50	50	107	8.36E − 10	1.24e − 11	15	8	6.25E − 11	3.30E − 12	11	8.59E − 11	4.54E − 12

^*a*^ Per site per generation.

Minimizing the effects of selection on mutations is essential for MA/WGS experiments to accurately reflect an organism’s spontaneous mutation rate and spectrum in a given environment. We accomplished this by repeatedly bottlenecking each MA line to reduce the N_e_, which we calculated as the harmonic mean of the number of cells at each generation between bottlenecks, assuming non-overlapping generations, a bottleneck size of one cell, and that every cell in every generation divides into two individuals. We determined that the N_e_ during MA was 11, 11, and 12 for *Lb. acidophilus, Lb. crispatus,* and *Lc. lactis*, respectively. As the minimum selection coefficient *s* required to overcome random drift is 1/N_e_ in haploid organisms (51), new mutations would need an extremely high absolute *s* of at least 0.09 to bias our results.

We performed multiple tests to determine if selection significantly biased the set of observed mutations during mutation accumulation. First, we tested whether the observed SNM, INS, and DEL counts in each species fit a negative binomial distribution, a generalization of the Poisson distribution that allows for greater variance. We found no evidence of deviation from the expected distribution (χ^2^ all *P* > 0.05) (Fig. S1). Second, we confirmed that SNMs within coding sequences of all three species did not occur more or less frequently than would be expected by chance (χ^2^ all *P* > 0.05).

### SNM spectra are heavily biased toward G:C → A:T

We observed high SNM rates in all three LAB species relative to previous MA/WGS experiments in other bacterial species, primarily driven by G:C → A:T transitions and suggesting oxidative damage as a potential cause of increased SNM rates (Fig. 2A). However, each species varied in the degree to which their mutation spectra were biased toward G:C → A:T transitions. In *Lb. acidophilus*, the rate of A:T → G:C transitions was only slightly lower than G:C → A:T transitions, resulting in an extremely high ratio of transition to transversion mutations (Ts/Tv) of 30.02, but a relatively low AT mutation bias (the ratio of the rate of SNMs converting GC sites to AT sites to the rate of SNMs converting AT sites to GC sites) of 2.97 (Fig. 2B). In contrast, the mutation spectrum of *Lb. crispatus* is much more balanced, as the rate of G:C → A:T mutations was only 11 times greater than its least frequent SNM. Thus, *Lb. crispatus* exhibited a Ts/Tv ratio of 1.50 and an AT mutation bias of 4.01, values well within the normal range for bacteria. Interestingly, *Lc. lactis* exhibits a relatively high frequency of G:C → T:A mutations and G:C → A:T mutations, leading to a greater AT mutation bias of 14.53 than would be expected given its genomic GC content of 35.24% and indicating a high fixation bias toward new GC alleles over AT alleles of 7.91 in *Lc. lactis*.

Additionally, we can compare the SNM rate and spectrum we observed in anaerobically cultured *Lc. lactis* to those observed in a prior MA/WGS study of *Lc. lactis* cultured in aerobic conditions (29). The largest change in the mutation spectrum between the two experiments was in G:C → T:A transversions, where the observed mutation rates were 3.6 times higher in aerobic than in anaerobic conditions (Fig. 2A).

### SNM rates in LAB are consistent with the drift-barrier hypothesis of mutation-rate evolution

To determine if the mutation rates of anaerobically cultured LAB conform to the predictions of the drift-barrier hypothesis of mutation-rate evolution, the same scaling law observed for aerobic species, we investigated the relationships between the mutation rates observed in this study and estimates of each species’ N_e_. We expect the N_e_ of the LAB species in this study to be relatively small due to their domestication for industrial use and host-associated lifestyles (40, 52). Using annotated, complete genome assemblies downloaded from RefSeq, we estimated the pairwise nucleotide diversity at fourfold synonymous sites (π) for each species. Assuming the relationship for haploid organisms, N_e_ = π/2μ, where μ is the per-site per-generation mutation rate (53), we estimate the N_e_ for *Lb. acidophilus* and *Lb. crispatus*, and *Lc. lactis* as 3.55 × 10^4^, 1.50 × 10^7^, and 1.01 × 10^7^, respectively. We observe the same qualitative reduction of diversity in *Lb. acidophilus* when comparing average nucleotide identity and gene content Jaccard distance within each species. Furthermore, we observe reduced genome sizes and fewer annotated protein-coding genes—signs of reduced N_e_ and relaxed selection in bacteria (40)—in *Lb. acidophilus* relative to the other two species (Fig. S2). These LAB N_e_ estimates are relatively small compared to the aerobic bacterial species previously evaluated with MA/WGS averaging ~1 × 10^8^ (12). For example, *Escherichia coli*, a species with a greater aerobic capacity and aerotolerance characteristic of its biphasic host/environment-associated lifestyle, has an estimated N_e_ ranging from 1.5 to 4.5 × 10^8^, which is ~10^4^ and ~10^1^ times larger than *Lb. acidophilus* and *Lb. crispatus*, respectively (40, 52, 54–56). Thus, while the mutation rates of anaerobically cultured LAB initially appear unexpectedly high, given these estimates of N_e_, our results are consistent with the drift-barrier hypothesis of mutation-rate evolution (55) (Fig. 2C).

### *Lb. acidophilus* ATCC 4356 lacks mismatch repair (MMR) and has a unique locational mutation bias

**Fig 3:**
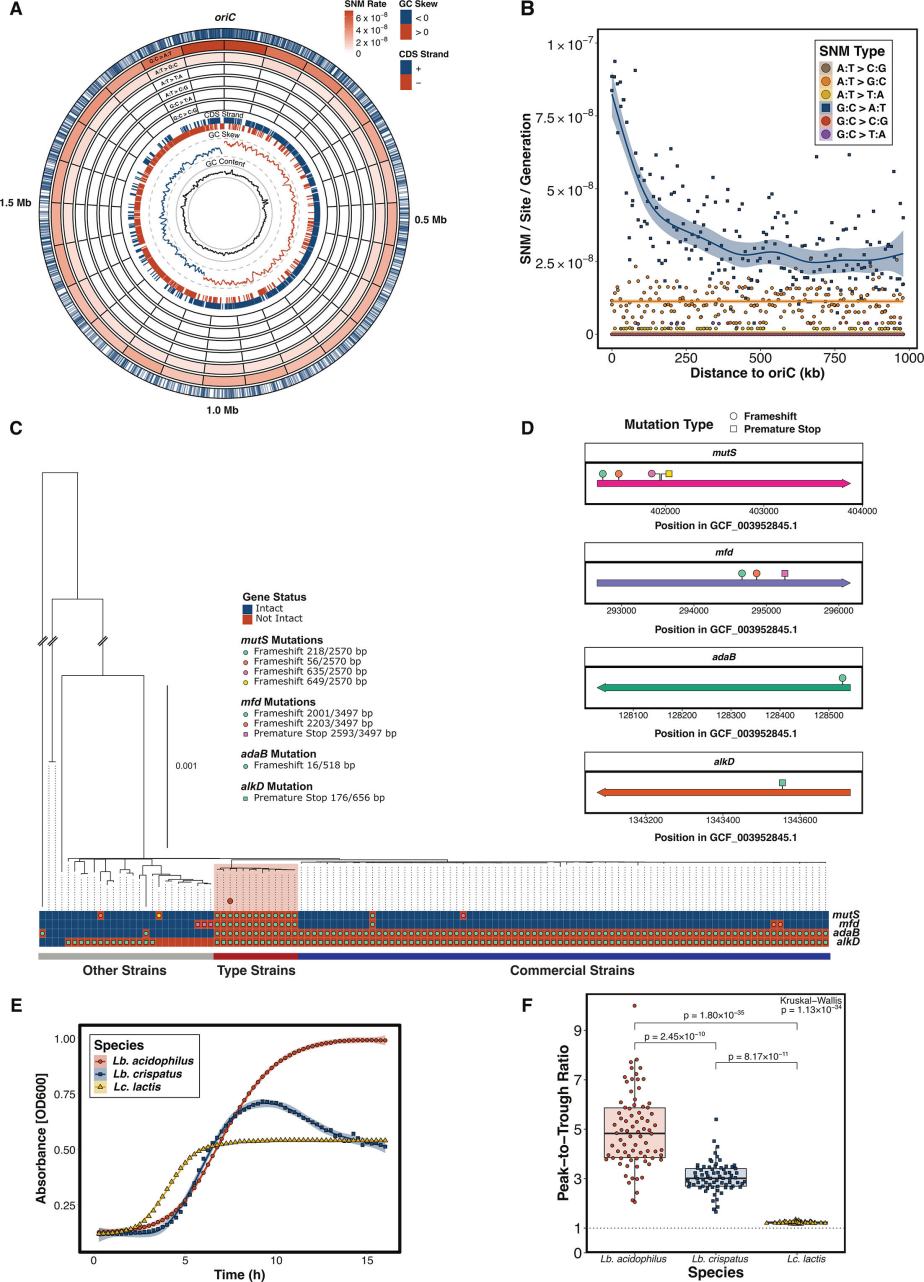
Potential causes and consequences of the elevated rate and divergent spectrum of mutations in *Lb. acidophilus.* (**A**) Circular genome plot of the *Lb. acidophilus* chromosome. From the outside in: labels indicating genomic coordinates in megabases, blue ticks showing the locations of all observed G:C → A:T transitions, heatmap showing the per-site per-generation rate of each of the six SNM types (from the outside in: G:C → A:T, A:T → G:C, A:T → T:A, A:T → C:G, G:C → T:A, and G:C → C:G) across 25 evenly sized (79,154 bp) bins, lines indicating the start positions of each CDS colored by strand, local (1,000 bp) GC skew colored by sign with dashed gray scale lines at 0.25 (outer) and −0.25 (inner), and local (1,000 bp) GC content with solid gray scale lines at 50% (outer) and 25% (inner). (**B**) Relationship between distance to the *oriC* and the per-site per-generation rate of each of the six SNM types across 10 kb bins. Square points represent G:C → A:T transitions, while circular points represent the other five SNM types. (**C**) Recombination-free maximum-likelihood phylogeny of 122 *Lb. acidophilus* genomes with the status of selected DNA repair genes. The *mutS*-deficient type-strains clade is highlighted in red. *Lb. acidophilus* ATCC 4365 is identified by the red circle. The scale bar represents 0.1 substitutions per site. Putative loss-of-function mutations that could be inferred for the DNA repair genes are overlaid as colored shapes. Strains are grouped into commercial, type, and other strains, as shown by the colored bar below. (**D**) Positions of putative loss-of-function mutations in the DNA repair genes using RefSeq assembly accession GCF_003952845.1, a strain with all four genes intact. (**E**) Growth curves of the ancestral MA strains in de Man, Rogosa, and Sharpe under anaerobic conditions. (**F**) Peak-to-trough ratios of the derived MA lines for each species. The Kruskal-Wallis *P*-value is in the top right corner, and the pairwise Bonferroni-corrected Dunn *post hoc P*-values are above the brackets.

Previous MA/WGS experiments conducted with genetically engineered mutator strains have identified variations in the mutation rate across the length of the bacterial chromosome, in which SNM rates peak approximately halfway between the origin of replication (*oriC*) and the replication terminus (33, 34, 36). To effectively detect locational biases in mutation rates, engineered mutator strains are typically required to generate a sufficient number of mutations to provide enough power to achieve statistical significance. Thus, the high SNM rate of *Lb. acidophilus* allowed us to screen for locational biases, revealing a ~3× increase in G:C → A:T transitions close to the *oriC* relative to the terminus (ρ = −0.53, Bonferroni-corrected *P* = 1.36 × 10^−15^) (Fig. 3A and B). Interestingly, this pattern is not even qualitatively present in the rest of the mutation spectra of *Lb. acidophilus,* nor in the mutation spectra of *Lb. crispatus* and *Lc. lactis* (Fig. S3, all |ρ| < 0.15, all Bonferroni-corrected *P* > 0.1).

Because these biases have been observed in engineered mutator strains but not in their wild-type counterparts, we sought to determine if *Lb. acidophilus* ATCC 4365—the species’ type strain used in our experiment—harbors mutations in DNA repair genes. Through pangenome profiling and phylogenetic inference using 122 annotated *Lb. acidophilus* assemblies from RefSeq, we confirmed that this species’ type strain has incurred a frameshift mutation in the *mutS* gene and therefore does not encode a functional MMR system (Fig. 3C and D). We found that the type strain’s entire lineage shares this mutation and that four distinct loss-of-function mutations in *mutS* have occurred across three lineages. The number of *Lb. acidophilus* strains lacking an intact copy contrasts with *Lb. crispatus*, where *mutS* was found to be intact in all 483 analyzed genomes (Fisher’s exact test *P* = 3.3 × 10^−12^). We found no evidence that the *Lb. crispatus* or *Lc. lactis* strains used in this experiment have DNA repair defects.

We observed the loss of other DNA repair genes across the *Lb. acidophilus* species tree: *adaB*, which encodes a DNA methyltransferase involved in the repair of O6-methylguanine residues (57, 58), and *alkD*, which encodes a DNA glycosylase that recognizes and excises alkylated nucleobases (59). Interestingly, most of the strains lacking *mutS* also lack the transcription-coupled repair protein *mfd* (Fig. 3C), a gene that, while associated with DNA repair, has also been shown to facilitate mutagenesis (60), especially in the presence of endogenous oxidative stress (61). An earlier genomic analysis noted separate lineages of “commercial sequences” (various dairy, probiotic, and commercial isolates) and “type strain sequences” (likely reisolated ATCC 4356 strains) within *Lb. acidophilus* (62). We observe a similar structure in our phylogeny of the species. The loss of *alkD* likely occurred before the emergence of the commercial and type-strain lineages, whereas *adaB* was lost in one strain with an exceptionally long branch and then again in the ancestor of the commercial and type-strain lineages, and *mfd* was separately lost in these lineages and elsewhere.

Initiation of replication requires the unwinding of DNA at the origin into single-stranded DNA, which is more susceptible to cytosine deamination (63). As such, we hypothesized that, in the absence of MMR, the elevated rates of G:C → A:T transitions near the origin in *Lb. acidophilus* may result from high rates of replication initiation. We assessed the possibility that *Lb. acidophilus* experiences more time undergoing replication than the other two species by performing growth curves cultivating the MA ancestor strains for each species in a microplate reader under anaerobic conditions. Our results show that *Lb. acidophilus* spends more time in logarithmic phase than *Lb. crispatus* and *Lc. lactis* (Fig. 3E). Furthermore, we investigated the sequencing coverage peak-to-trough ratio. Because of the directional nature of bacterial replication, sequencing coverage is expected to peak at the replication origin and decline along the genome toward the replication terminus. A higher ratio of sequencing coverage at the replication origin relative to the terminus suggests increased replication initiation rates and greater exposure of DNA in a single-stranded state near the origin. To quantify this for each MA line, we used iRep (64) with the same sequencing data used to call the mutations as input. We found that the mean peak-to-trough ratio of *Lb. acidophilus* is significantly higher than that of *Lb. crispatus* and *Lc. lactis* (Fig. 3F, Kruskal-Wallis *P* < 10^−33^, Dunn *post hoc* all *P* < 10^−9^).

### Small indel mutations are also elevated in LAB

**Fig 4:**
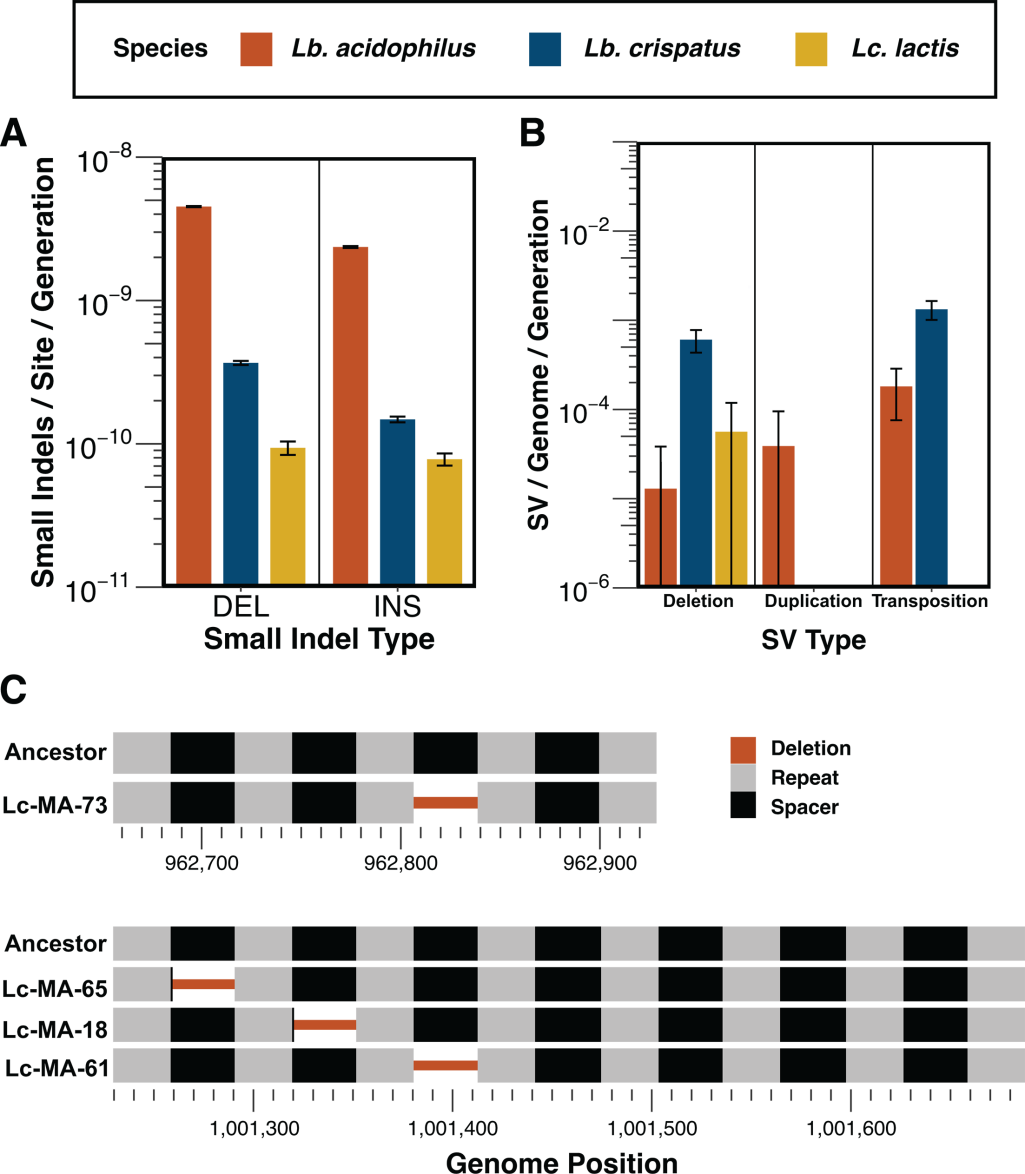
Rates of indels and SVs, as well as the locations of deleted clustered, regularly interspaced, short palindromic repeat (CRISPR) spacers in *Lb. crispatus.* (**A**) Per-site per-generation rates of small (<50 bp) indels. Error bars represent 95% confidence intervals. (**B**) Per-genome per-generation rates of SVs. Error bars represent 95% confidence intervals. Bars reaching the bottom of the plot fell below 0. (**C**) Locations of CRISPR spacer deletions at the two arrays in *Lb. crispatus*. Thick lines represent intact repeat (gray) and spacer (black) sequences. Thin red lines represent deleted regions.

In addition to elevated SNM rates, anaerobically cultured LAB had high rates of small (<50 bp) indels. In total, we observed 1,042 indels in *Lb. acidophilus*, 71 in *Lb. crispatus*, and 19 in *Lc. lactis*, resulting in respective rates of 6.82 × 10^−9^ (SE: 3.18 × 10^−11^), 4.57 × 10^−10^ (SE: 6.50 × 10^−12^), and 1.48 × 10^−10^ (SE: 5.21 × 10^−12^) indels per site per generation (Table 1; Fig. 4A). The average length of insertions was 1.0 (SE: 0), 9.9 (SE: 2.8), and 5.5 (SE: 2.7) nucleotides in *Lb. acidophilus*, *Lb. crispatus*, and *Lc. lactis*, respectively, while the average deletion lengths were 1.5 (SE: 0.2), 21.4 (SE: 3.4), and 3.3 (SE: 1.7) nucleotides. Similar to their SNM rates, these results place LAB on the high end of studied bacterial indel rates but are in line with previous MA/WGS results in *Lc. lactis* (1.14 × 10^−10^) (29), *S. pneumoniae* (8.9 × 10^−10^) (13), and *S. suis* (2.39 × 10^−10^) (30). All three species had higher rates of small deletions than insertions, which is a commonly observed phenomenon across bacteria (65).

### Insertion sequence (IS) elements were active in the Lactobacilli but not *Lc. lactis*

A previous MA/WGS experiment revealed an increased transposition rate of IS elements in anaerobically cultured *E. coli* relative to aerobically grown controls (14). To determine if anaerobically cultured LAB similarly exhibit elevated IS element transposition rates compared to aerobically cultured bacteria, we profiled SVs with GRASPER (66). While our estimates of structural variant mutation rates are conservative due to the limitations of short-read sequencing, we identified 155 SVs that arose during the MA: 18 in *Lb. acidophilus*, 134 in *Lb. crispatus*, and 3 in *Lc. lactis* (Fig. 4B) (Data set S3). Most SVs observed in the Lactobacilli were transpositions caused by IS elements, whereas all three SVs found in *Lc. lactis* were deletions. In *Lb. crispatus*, 92 (69%) of SVs were transpositions caused by IS elements, with 87 (95%) caused by IS256 family transposons. The observed rates of IS element transposition in the Lactobacilli are comparable to other aerobically cultured bacteria, as rates of 2.10 × 10^−4^ and 2.50 × 10^−3^ transpositions per genome per generation were previously reported for *Burkholderia cenocepacia* and *Deinococcus radiodurans*, respectively (67) (Fig. 4B).

### Clustered, regularly interspaced, short palindromic repeat (CRISPR) spacers are deletion hotspots in *Lb. crispatus*

CRISPR arrays are part of a bacterial adaptive immune system (68). CRISPR loci consist of short repeat sequences that flank spacer sequences commonly derived from infectious mobile elements, such as phages. These spacer sequences serve as a molecular memory and provide the capability to resist infection by parasites that have previously infected an individual’s ancestor. We observed four distinct spacer sequence deletions across the two annotated CRISPR loci in *Lb. crispatus* (Fig. 4C). In two cases, the deletion left behind a single base pair of the spacer, while in the other two cases, the deletion removed the entire spacer. While the exact mechanism behind these mutations is unknown, it is unlikely that these deletions are due to misalignment during replication because both flanking repeats were left intact (69).

## DISCUSSION

LAB are foundational members of the human microbiome and are crucial for numerous industrial processes (23, 41, 70, 71). Additionally, their facultative to aerotolerant anaerobic physiology makes them ideal candidates for studying how oxygen-tolerant lifestyles affect mutation rates. Here, we report the results of a mutation accumulation experiment performed for over 1,000 generations across at least 50 replicate lines from each of three industrially and biomedically important LAB species. Our study reveals exceptionally high mutation rates and strongly biased spectra in *Lb. acidophilus*, *Lb. crispatus*, and *Lc. lactis* relative to the aerobic bacterial species investigated in previous MA/WGS studies. Moreover, these findings align with results from MA/WGS studies of the aerobically cultured LAB *Lc. lactis* (29), *S. pneumoniae* (13), *S. suis* (30), and the anaerobically cultured archaeon *T. eurythermalis* (15), suggesting that reduced oxygen tolerance may be associated with increased mutation rates, even in minimal oxygen environments. As pairwise diversity estimates from whole-genome and MLST data suggest these LAB species represent smaller N_e_ stemming from their domesticated and host-associated lifestyles (12, 40, 52, 62, 72–75), our results are consistent with the drift-barrier hypothesis of mutation-rate evolution (55). These results highlight the importance of neutral processes in driving prokaryotic genome evolution, especially in host-adapted and domesticated organisms with small N_e_.

One factor possibly contributing to the higher mutation rates observed in LAB species is their characteristic peroxide production, attributed to their highly reactive enzymes, including pyruvate oxidase, lactate oxidase, NADH oxidase, and NADH-dependent flavin reductase (25, 76, 77), and the minimal presence of superoxide-scavenging proteins encoded in their genomes (Fig. 1). Unlike *E. coli*, a facultative anaerobe that thrives in aerobic conditions and has a mutation rate approximately 90-fold lower than *Lb. acidophilus*, LAB species typically lack catalase (*katE*/*katG*) and superoxide dismutase (*sodA*/*sodC*) genes—two key enzymes responsible for scavenging endogenously produced peroxide and oxygen radicals. We observe strongly biased mutation spectra in LAB, with G:C → A:T transitions representing the majority of SNMs. Although oxidative damage is associated with increased rates of G:C → A:T mutations, prior investigations suggest that LAB produce only trace amounts of H_2_O_2_ under hypoxic and anaerobic conditions (77, 78). As such, either LAB are sensitive to the minimal oxygen conditions of the anaerobic chamber (~20 ppm of O_2_) or other factors contribute to G:C → A:T bias in anaerobic conditions.

Supporting the possibility that causes other than oxidative damage can produce a G:C → A:T-biased mutation spectrum is the observation of a similar G:C → A:T bias when assessing the anaerobic mutation rates of *E. coli* (14). Here, Shewaramani et al. report almost identical G:C → A:T mutation rates in aerobic and anaerobic conditions when cultivating their *E. coli* MA lines on minimal-glucose media, which can promote fermentation in aerobic and anaerobic atmospheres. Fermentative metabolism characteristically produces organic-acid byproducts, and acidic pH can promote DNA damage through protonating cytosine and adenine, increasing the potential for deamination (79). Given that the cultivation of our LAB MA lines is similarly fermentative and that the back mutation of A:T → G:C also occurs at an elevated rate, if not nearly identical, in *Lb. acidophilus*, spontaneous deamination may be a major contributor to the enhanced mutation rates of LAB. However, the high A:T → G:C transition rate of *Lb. acidophilus* may also be due to the type strain’s lack of a functional MMR system, a pattern observed in previous MA/WGS experiments (13, 49, 80).

In addition to its distinct mutation spectrum, we observe an unexpected pattern regarding the chromosomal distribution of G:C → A:T transitions in *Lb. acidophilus*, in which the G:C → A:T transition rate is ~3 times higher in the 250 kb flanking the replication origin compared to the rest of the genome. Previous studies investigating genetically engineered mutator strains have identified genome position-dependent SNM rate variation (33, 34, 36). However, rather than mutation rates peaking in the region flanking the origin, these studies describe a peak in the mutation rate mid-chromosome, between the replication origin and terminus. Alternatively, in eukaryotes, high mutation rates have been reported near specific human replication origins (81). In these cases, multiple SNM types drive the increased mutation rates, with the cause attributed to mechanisms that predominantly affect the immediate vicinity of the origin, such as abortive topoisomerase activity (81).

Since G:C → A:T transitions exclusively drive the mutational enrichment in *Lb. acidophilus,* and the enrichment extends significantly further from the origin (~250 kilobases beyond the *oriC* in *Lb. acidophilus* compared to ~2.5 kilobases in humans), we hypothesize that this enrichment may arise from a distinct underlying mechanism: the unique vulnerability of single-stranded DNA in the prokaryotic replichore (63). In the absence of MMR, this effect is likely amplified in *Lb. acidophilus* due to its higher replication initiation rates and prolonged time spent in the logarithmic growth phase during MA, compared to *Lb. crispatus* and *Lc. lactis*. Further investigation will be required to fully understand the mechanism behind this unique locational bias and the extent to which it may be dependent on the strain or environment.

In contrast to the unique positional mutation bias observed in *Lb. acidophilus*, *Lc. lactis* exhibits an exceptionally strong AT mutation bias. While an AT-biased mutation spectrum is not uncommon in prokaryotes (82), the bias in *Lc. lactis* is unusually pronounced, with an equilibrium GC content of just 8% (Fig. 2B), significantly lower than its genomic GC content of 35%. Given this discrepancy, factors beyond mutation must contribute to maintaining the genomic GC content, such as GC-biased gene conversion and selection. GC-biased gene conversion is most commonly discussed in diploid eukaryotes and can maintain genomic GC content by favoring GC alleles when resolving DNA heteroduplexes formed during homologous recombination (83). However, the low rate of homologous recombination in *Lc. lactis* suggests that the impact, if any, of gene conversion on genome composition is minimal, even if compositionally biased (84). Alternatively, selection can also influence genome composition, with factors such as nitrogen availability (85), exposure to oxygen (86), temperature (87), and codon usage (88) serving as possible sources of selective pressure. The minimum GC content required to encode all 20 amino acids is estimated to be around 20%–25% (88), significantly higher than the 6.4% equilibrium GC content predicted for *Lc. lactis*. While some bacterial genomes have GC contents as low as 15%, these are typically obligate host-associated species (89, 90). This suggests that selective pressure to preserve a functional coding genome likely plays a key role in maintaining *Lc. lactis*’s 35% GC content.

A previous MA/WGS experiment performed on aerobically cultured *Lc. lactis* allows us to compare mutation spectra across conditions (29). We note that the largest difference in the mutation spectrum is in G:C → T:A transversions, which occurred aerobically at a rate 3.6 times greater than they did anaerobically (Fig. 2A). As cytosines are particularly susceptible to mutation due to oxidative damage through 8-oxoguanine formation, which can result in mismatches with adenine (27, 28), these results are consistent with the hypothesis that the biased mutation spectrum of LAB is due to oxidative damage.

*Lb. crispatus* also exhibits unique mutational characteristics. In addition to its elevated SNM rates, we identified four independent deletions within its CRISPR loci (Fig. 4C). These deletions removed most or all of a CRISPR spacer while leaving the surrounding repeats intact. CRISPR activity is particularly significant in LAB, as foundational discoveries describing CRISPR-mediated phage defense were first made in *Streptococcus thermophilus*, a key species in yogurt and cheese production (91). Our literature search revealed examples where both the spacer and its adjacent tandem repeat were deleted (92–96), but we found no reports of deletions affecting only the spacer. It is possible that losing only the spacer could disrupt CRISPR RNA maturation or interfere with the incorporation of new spacers into the array. Either scenario could weaken the bacterium’s defense against genomic parasites, making such deletions subject to strong negative selection in both wild and industrial strains. Alternatively, our findings suggest that CRISPR locus rearrangements may occur through a multi-step process, beginning with the loss of a spacer, which then increases the likelihood of losing the adjacent repeat. In this context, the *Lb. crispatus* ATCC 33820 strain could serve as a valuable model for studying the mechanisms that maintain CRISPR arrays.

Lastly, we identified multiple losses of *mutS* in *Lb. acidophilus*, including in the lineage containing the type strain ATCC 4356. Given this species’ low N_e_, the persistence of MMR deficiency indicates weak selection against mutator alleles, in line with the drift-barrier hypothesis of mutation-rate evolution (55). The elevated SNM rate observed in the MMR-deficient type strain further supports this framework (Fig. 2C), suggesting that, as predicted by the drift-barrier hypothesis, *Lb. acidophilus* is approaching the mutation-rate equilibrium shaped by its domestication history.

It is intriguing to consider why *Lb. acidophilus* harbors such low genetic diversity. As a widely used dairy fermenter and probiotic, many strains are subject to repeated artificial selection and severe bottlenecks during industrial propagation. While the consequences of domestication and artificial selection are well documented in plants and animals, studies of domesticated microbes likewise reveal reduced genetic diversity (97) and the emergence of MMR-deficient lineages (98). Given that loss of DNA repair genes is pervasive in *Lb. acidophilus*—especially in the commercial and type-strain lineages—this species may provide a powerful model for studying mutation-rate evolution and the genomic consequences of microbial domestication in real time, highlighting the need for deeper investigation into its population biology.

In conclusion, the MA/WGS method has proven to be a powerful tool for characterizing mutation rates and patterns across the tree of life. To fully understand how mutation rates shape and are shaped by evolution, it is essential to consider the environmental contexts in which they arise. Here, by analyzing the high mutation rates of aerotolerant anaerobes under anaerobic conditions, we provide further evidence that the mutation dynamics of anaerobic species differ from those of organisms adapted to atmospheric oxygen levels while still aligning with the drift-barrier hypothesis of mutation-rate evolution. We also demonstrate an abundance of mutations associated with spontaneous deamination, highlighting the inherent fragility of purines even under conditions that minimize oxidative stress. Given these high mutation rates, careful handling when maintaining lactic acid bacteria cultures is crucial in laboratory and industrial settings. Our comparisons to aerobically cultured *Lc. lactis* suggest that exposure to oxygen-rich conditions can accelerate mutation rates, potentially leading to unintended consequences for stock populations. Ultimately, our work illustrates the importance of studying mutation rates in fastidious microbes less adapted to aerobic conditions and highlights the role of non-adaptive evolutionary mechanisms, including demography and drift in bacteria. As aerotolerant and obligate anaerobes represent a significant fraction of the human microbiome and include key species in food and bioenergy production, further investigation into the mutation properties of these taxa is essential.

## MATERIALS AND METHODS

### Bacterial strains

We acquired the three strains that served as ancestors for mutation accumulation: *Lb. acidophilus* ATCC 4356, *Lb. crispatus* ATCC 33820, and *Lc. lactis subsp. lactis* ATCC 11454, directly from the American Type Culture Collection (ATCC). Upon receipt, the strains were immediately cultivated as directed by resuspending the entire lyophilized pellet in 0.5 mL of de Man, Rogosa, and Sharpe (MRS) culture media (Remel R454062) and incubating in 95% air, 5% CO_2_ at 37°C until turbid. Cultures were then vortexed and streaked for isolation on MRS agar (Fisher Scientific, BP9744-5), before cryogenically storing stock cultures in 40% glycerol at −80°C.

### Construction of phylogeny and identification of oxidative stress-associated genes

The reference genomes for our MA ancestor strains (*Lb. acidophilus* ATCC genome ID: 4744cca046d94f76, *Lb. crispatus* NCBI accession: NZ_CP072197.1, and *Lc. lactis* ATCC genome ID: 5635357f23ad4cd3) and for *S. pneumoniae* (NCBI accession: NC_008533.2), *S. suis* (NCBI accessions: PRJNA763404), *E. coli* (NCBI accession: NC_000913.3), and *T. eurythermalis* (NCBI accession: CP008887.1) were annotated with the EggNOG-mapper v.2 web tool using default settings (99). To build the phylogenetic tree, sequences of the *rpoB* gene from each species (*S. suis* MA2 was used as a representative for its species) were aligned with MAFFT v.7.505 using the auto option (100). IQ-TREE v.2.2.2.6 was used to select the best model and infer the maximum-likelihood phylogeny (101, 102). Annotations were then manually profiled for genes previously reviewed as related to oxidative stress in the genus *Lactobacillus* (25). The phylogeny was visualized in R with ggtree 3.16.2 (103).

### MA culture conditions

To initiate MA, all three bacterial strains were revived in anaerobic conditions (90% N_2_, 5% CO_2_, 5% H_2_; vinyl anaerobic chamber, Coy Lab) by streaking for isolation on MRS agar. Plates were incubated anaerobically at 37°C by transferring the inoculated plates into an anaerobic jar (BD, 260622) containing an anaerobic atmosphere-generating pack (Thermo, R681001). Anaerobic jars containing plates were only opened within the anaerobic chamber and sealed before removal from the anaerobic chamber. After 48 h of incubation, we randomly selected 96 isolated colonies from each of the revived ancestor plates and restreaked these colonies for isolation on MRS agar, with each MA line occupying a single quadrant of a four-quadrant Petri dish (VWR, U25384-348). To ensure random selection of colonies and reduce the possibility of selection bias in colony picking, each plate quadrant was pre-dotted about 0.5 inches from the plate intersection. Lines were subjected to single-cell bottlenecks every 48 h by selecting the colony closest to the dot and restreaking for isolation over the course of 100 days, resulting in 50 transfers. Every 10 transfers, the colony second closest to the dot was selected as a representative colony and was stored for historical record in 96-well plates containing 40% glycerol at −80°C. After the final transfer, the colony closest to the dot was resuspended in a 96-well plate containing 40% glycerol and stored at −80°C.

### Estimating MA generations

We used a standard protocol to estimate the number of MA generations for each species (35, 104, 105). Briefly, we obtained single colonies of the MA ancestor of each species by streaking for isolation on MRS agar and incubating anaerobically for 48 h at 37°C. Plates were imaged, and the area of five random colonies was quantified using ImageJ (106). The colonies whose area was quantified were then suspended and plated to determine the respective number of colony-forming units (CFU). This process was repeated every 10 transfers to track the average colony population size throughout the MA experiment. Using the average colony population size in CFU at each of the five time points, we calculated the number of generations in each 48 h incubation period as the average of the number of generations, *g*, estimated for each time point using the following equation: $g=\frac{log(CFU)}{log(2)}$.

### DNA extraction and sequencing

At the conclusion of the MA experiment, each MA line and the ancestral strain for each species was struck a final time onto MRS agar and incubated anaerobically for 48 h at 37°C. A loop of cells was collected across the plate to ensure the collection of an appropriate amount of biomass and that no new novel mutation would have been fixed during the collection of cells for DNA sequencing. DNA was then extracted with the Qiagen DNeasy UltraClean Microbial Kit (Qiagen, 12224-250), with lysis steps completed under anaerobic conditions. A quality control analysis was performed on the DNA samples using the Qubit HS-dsDNA quantification kit (Qubit, Q32854) to determine quantity before submission to the VANTAGE core for library preparation and DNA sequencing. The samples were normalized to target 100 ng of total input for each sample, and libraries were prepared using the Twist Biosciences kit (Twist, 104207) following a miniaturized version of the manufacturer’s protocol. The libraries were quantitated and pooled for downstream processing. The pool quality was assessed using the Agilent Bioanalyzer and quantified using a qPCR-based method with the KAPA Library Quantification Kit (Kapa, KK4873) and the QuantStudio 12K instrument. Prepared libraries were pooled in equimolar ratios, and the resulting pool was subjected to cluster generation using the NovaSeq 6000 System, following the manufacturer’s protocols. One hundred fifty base pair paired-end sequencing was performed on the NovaSeq 6000 platform, targeting 2M reads per library. Raw sequencing data (FASTQ files) obtained from the NovaSeq 6000 were subjected to quality control analysis, including read quality assessment. Real-Time Analysis Software and NovaSeq Control Software (1.8.0; Illumina) were used for base calling. MultiQC (107) (v.1.7; Illumina) was used for data quality assessments.

### Quality control and mutation identification

Raw sequence reads were trimmed and filtered with fastp 0.23.4 (108). Processed reads were then checked for contamination with Kraken2 2.1.3 (109). The processed reads from uncontaminated samples were then aligned to their respective reference genomes using BWA 0.7.10-r789 (110). Alignment metrics were generated with Samtools 1.20 (111). Mean sequencing depth for analyzed samples was 110, 221, and 88 for *Lb. acidophilus*, *Lb. crispatus*, and *Lc. lactis*, respectively. Mean sequencing coverage for analyzed samples was 99.99, 99.1, and 99.87 for *Lb. acidophilus*, *Lb. crispatus*, and *Lc. lactis*, respectively (Data set S1). Raw and processed sequence reads were analyzed with FastQC 0.12.1 (https://github.com/s-andrews/FastQC), and these results, along with the Kraken2 output and alignment statistics, were visualized with MultiQC 1.19 (107). Duplicate reads in samples without contamination and with sufficient mapped read depth were identified with Picard Tools, and then SNM and small indel variants were identified with the HaplotypeCaller program in GATK 4.1.2.0 (112) with ploidy set to 1. Variant calls from GATK were hard filtered with BCFtools 1.18 (111) using the following filters: F_MISSING = 0, QD ≥ 2, SOR ≤ 5, FS ≤ 60, MQ ≥ 40, MQRankSum ≥ −20, ReadPosRankSum ≥ −8, and QUAL ≥ 30. Five ancestral differences in *Lb. acidophilus*, one in *Lb. crispatus*, and one in *Lc. lactis* were removed. Only chromosomal variants were considered, so variants called on the plasmids present in *Lc. lactis* were removed. Lines were then manually checked and filtered for cross-contamination. Mutations occurring in close proximity (<50 bp) to one another within the same MA line are likely not caused by independent mutational events, so these mutations were omitted from downstream analyses as has been done previously (35, 113). Larger structural variants were identified with GRASPER 0.1.1 (66). Structural variants were manually quality-controlled using the Integrative Genomics Viewer 2.15.2 (114). To fully annotate the deletions observed in the CRISPR arrays of *Lc. crispatus*, we used the specialized annotation web tool CRISPRCasFinder 4.2.30 with default options (115). A full list of small and large mutations can be found in Data sets S2 and S3, respectively.

### Mutation rate and spectrum calculations

For each MA line, the SNM and small indel rates were calculated using the formula μ=m/nT, where *μ* is the mutation rate per site per generation, *m* is the number of observed mutations, *n* is the number of sites analyzed (total length of the ancestral chromosome), and *T* is the number of MA generations for that species. The SNM spectrum was calculated using the same formula, with *m* being the number of observed mutations of a given type and *n* being the number of sites where that type of mutation could occur. Rates of SVs per genome per generation were calculated for each line using the formula μ=m/T, where *m* is the number of SVs observed in a line. For each mutation type, the rate for the species was calculated as the mean of the rates for each MA line in that species. Standard errors for mutation rates in each species were calculated using the formula $SE=s/\sqrt{N}$, where *s* is the standard deviation of mutation rates among the MA lines and *N* is the number of MA lines. Ninety-five percent confidence intervals were calculated by multiplying the SE by 1.96. Mutation rates for each line can be found in Data set S1. Rates for each SNM type in the bins analyzed in Fig. 3A and B were calculated using the formula μ=m/nTl, where *m* is the total number of that SNM type observed within the bin for that species, *n* is the number of sites where that SNM type could occur in that bin, and *l* is the number of MA lines for that species.

### Mutation bias calculations

The AT mutation bias (*β*) was calculated using the formula β=(GC → AT)/(AT → GC), where *β* is equal to the ratio of GC → AT mutations to AT → GC mutations. To calculate fixation bias, we first calculated the equilibrium genome-wide AT composition (*α*_eq_) that would be expected under neutral evolution from *β* using the formula $\alpha_{eq}=\beta /\left(1+\beta \right)$ (12). When selection acts on genome composition, *α*_eq_ can be estimated with the formula $\alpha_{eq}\approx \beta \varphi /\left(1+\beta \varphi \right)$, where *φ* is the fixation bias, or ratio of the rate of fixation for new AT alleles relative to that of new GC alleles (12, 51, 116). To estimate *φ*, the formula can be rewritten as $\varphi \approx \alpha_{obs}/\left(\beta -\beta \alpha_{obs}\right)$, where *α*_obs_ is the observed genome-wide AT composition.

### Effective population size estimates

To estimate the N_e_ for each of the three species, we downloaded all available complete, annotated genome assemblies from the RefSeq database (117). We confirmed that each genome was isolated from a member of the correct species using ConSpeciFix (118), and we removed duplicate assemblies of the same strain based on the “Organism Infraspecific Names Strain” column of the assembly data reports. We then used Panaroo 1.5.2 (119) to profile the pangenome of each species. Codon alignments for single-copy genes present in all samples within each species were generated by extracting the gene sequences from the genome using the coordinates produced by Panaroo with bedtools 2.31.1 (120), translating the sequences with the Python library egglib 3.5.2 (121), aligning the protein sequences with MAFFT 7.525 (100), and threading the DNA sequences onto the protein alignments with the Phykit 2.0.2 “thread_dna” function (122). We then calculated population genetic statistics, including π (123), within fourfold synonymous sites using the egglib library. Using the relationship $N_{e}=\pi /2\mu$, we then calculated N_e_. Gene content Jaccard distances were calculated with vegan 2.7-1 (vegandevs.github.io/vegan/). Average nucleotide identity was calculated with fastANI 1.34 (124).

### External mutation data

To illustrate the relationships between mutational parameters in Fig. 2B and C, we used a publicly available collection of mutation data (12). Data for *Mycoplasma mycoides* JCBI-syn1.0 was removed, as this is a heavily engineered mutant strain. Data for species with multiple measurements were averaged. To visualize the difference between the SNM rates that were observed and those that would be expected based on the drift-barrier hypothesis for the three anaerobically cultured LAB, we generated a linear regression model using the prokaryotic N_e_ and SNM rate data while excluding the new LAB results. This model was then used to predict the SNM rates of the three anaerobically cultured LAB from their estimated N_e_.

### Examining the relationship between proximity to the *oriC* and the mutation spectrum

The location of the *oriC* was identified using Ori-Finder 2022 with default options (125). The relationship between the distance from the start of the bin to the *oriC* and the rate of each SNM type for each species was tested using Spearman correlation with Bonferroni *P*-value correction. Peak-to-trough ratios for each MA line were calculated using the bPTR function in iRep 1.1.14 (64) with the same BAM files used to call mutations. For the growth curves, the ancestral strains of each species were struck out on MRS agar plates and cultured as described in the MA protocol above. Three colonies per species were inoculated into liquid MRS and incubated overnight, shaking at 37°C. From these cultures, 1.5 µL were inoculated into 150 µL of liquid MRS in a 96-well plate. The growth curve was then performed in an Agilent BioTek Epoch 2 plate reader, shaking at 37°C, with the optical density at 600 nm measured every 15 minutes.

### *Lactobacillus* evolutionary genomics

To produce Fig. 3C and D, we downloaded all available annotated assemblies from RefSeq for *Lb. acidophilus* while excluding metagenome assemblies and duplicate strains (117). Species membership was confirmed with ConSpeciFix 1.3.0 (118). To reconstruct the recombination-filtered phylogeny, a core-genome alignment was generated with Parsnp 1.2 (126) using the “-c” flag to force the inclusion of every assembly. The resulting xmfa file was converted to multi-FASTA format with Harvesttools 1.1.2 (126) and then input into ClonalFrameML 1.13 (127) with the “-emsim” flag set to 1,000 to account for uncertainty in the estimated parameters, which produced a recombination-filtered alignment. Finally, this recombination-filtered core-genome alignment was used to reconstruct the species phylogeny with IQ-TREE 2.4.0 (102) run in “safe” mode. Model selection was performed with ModelFinder (101), and branch supports were calculated using 1,000 nearest-neighbor interchange-optimized ultrafast bootstrap (128) replicates. The phylogeny was visualized in R with ggtree 3.16.2 (103).

Pangenome profiling was performed with Panaroo 1.5.2 with the clean mode and refind mode options set to strict (119). To compare the prevalence of the *mutS* gene between *Lb. acidophilus* and *Lb. crispatus*, all annotated, non-metagenome-assembled genomes were downloaded from RefSeq and profiled with Panaroo with the same settings. Given that gene presence inference can be complicated by assembly artifacts, we sought to more thoroughly evaluate the status of the genes featured in Fig. 3C and D. We performed a search for the Panaroo-selected reference sequence of each of these genes in each assembly using blastn 2.17.0+ (129). For each gene and assembly, we extracted the range of the blastn hits, if present, as well as the gene as annotated by Panaroo, if present, with bedtools 2.31.1 (120). These ranges were merged, and the sequences were extracted. Gene sequences split between contigs were manually reconstructed using substantial overlap within the fragmented gene sequences. These sequences were then aligned with MAFFT 7.5.25 (100) and assessed for loss-of-function mutations, including indels causing frameshifts and SNMs resulting in premature stop codons.

## Data Availability

All code for data analysis and production of figures can be found on the Behringer Lab’s GitHub at github.com/BehringerLab/LAB_MA_2025. Whole-genome sequencing data generated during this study are publicly available on SRA at NCBI BioProject PRJNA1228167.

## SUPPLEMENTAL MATERIAL

Data set S1 mbio.03054-25-s0001.xls Per line summary of mutation accumulation results.

Data set S2 mbio.03054-25-s0002.xls Complete list of SNMs and small structural variants.

Data set S3 mbio.03054-25-s0003.xls Observed large structural variants.

Supplemental Material mbio.03054-25-s0004.docx Table of contents for supplemental files and Figures S1-S3.

ASM does not own the copyrights to Supplemental Material that may be linked to, or accessed through, an article. The authors have granted ASM a non-exclusive, world-wide license to publish the Supplemental Material files. Please contact the corresponding author directly for reuse.
