# Supplementary material for: Elevated rates and biased spectra of mutations in anaerobically cultured lactic acid bacteria
Source: mBio. 2025 Nov 13;16(12):e03054-25. doi: 10.1128/mbio.03054-25 (PMC12691593; doi:10.1128/mbio.03054-25)
Supplement: Supplemental Material — Table of contents for supplemental files and Figures S1-S3. [file mbio.03054-25-s0004.docx]

Supplementary Materials for:

**Elevated rates and biased spectra of mutations in anaerobically cultured lactic acid bacteria**

Owen F. Hale, Michelle Yin, and Megan G. Behringer

Table of Contents:

Supplementary Figure S1: Fitting of SNM counts against a negative binomial distribution.

Supplementary Figure S2: Comparison of genomic assembly and diversity metrics between species.

Supplementary Figure S3: Relationship between distance to the oriC and the per site per generation SNM rate.

Supplementary Dataset S1: Per Line Summary of Mutation Accumulation Results

Supplementary Dataset S2: Complete List of SNMs and Small Structural Variants

Supplementary Dataset S3: Observed Large Structural Variants


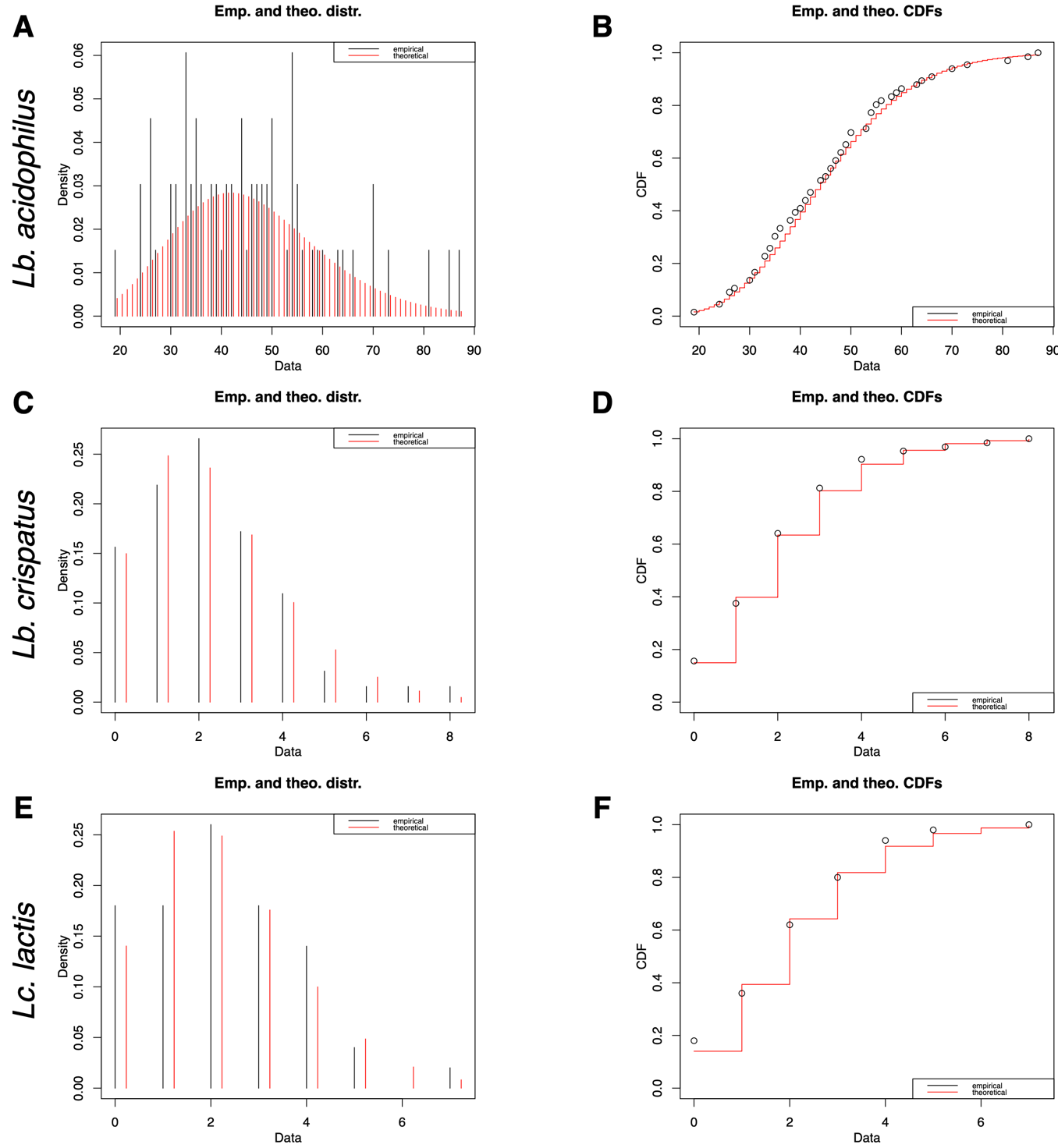


**Figure S1. SNM counts fit a negative binomial distribution.** Probability density (A, C, and E) and cumulative density (B, D, and F) plots of observed (black) and expected (red) mutation counts given a negative binomial distribution with parameters fit by the R package fitdistrplus.

**Figure S2. Comparison of genomic assembly and diversity metrics between species.** Number of annotated protein coding genes (A) and assembly length (B) of complete RefSeq assemblies for each species. Intraspecific diversity measured by average nucleotide identity (C) and gene content Jaccard distance between complete RefSeq assemblies for each species. All comparisons within each metric were assessed for statistical significance with a Kruskal-Wallis test followed by a Dunn post-hoc test with Bonferroni correction. All p < 0.01.


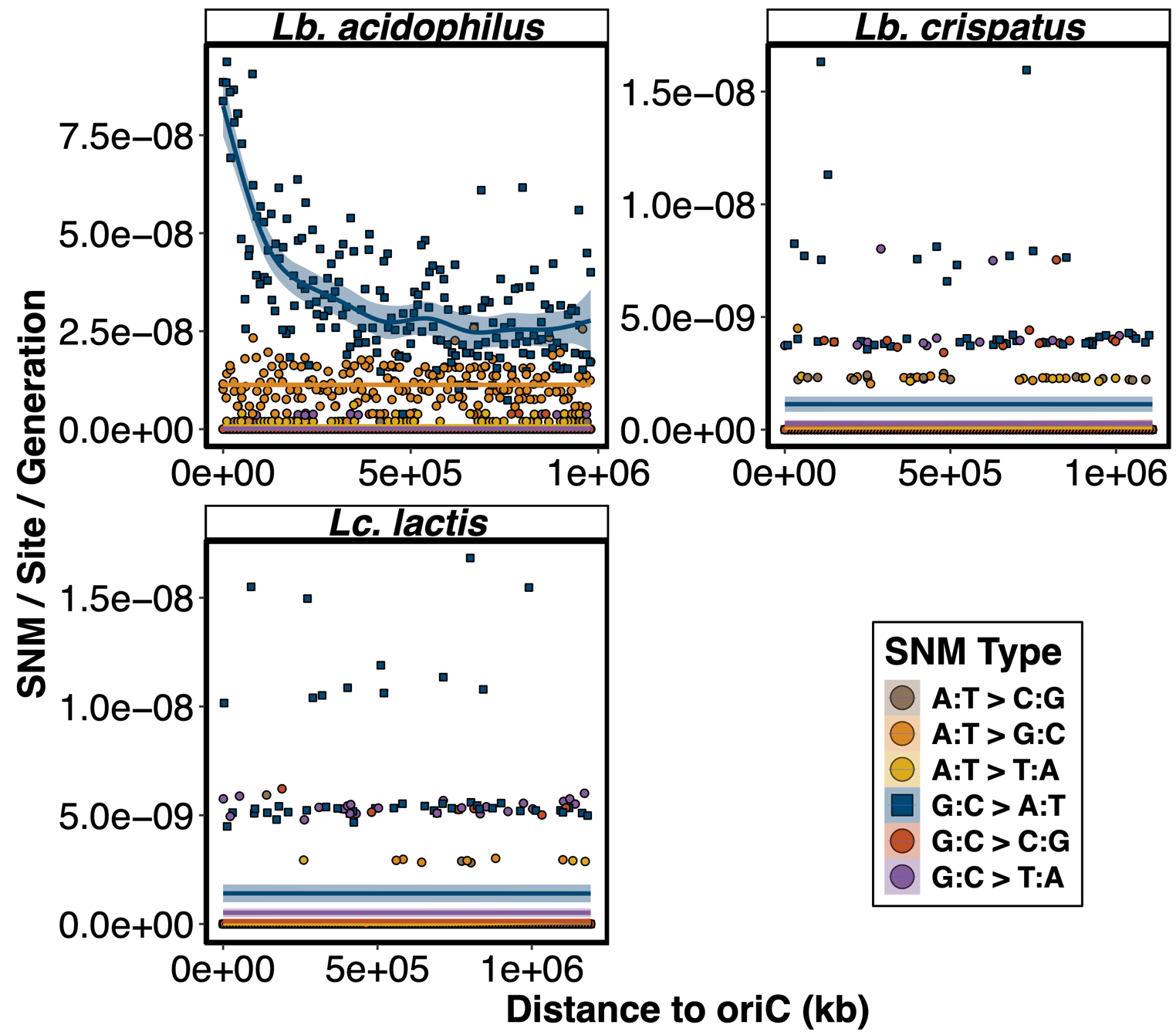


**Figure S3. Relationship between distance to the oriC and the per site per generation rate of each of the 6 SNM types across 10 kb bins in all three species.** Square points represent G:C → A:T transitions while circular points represent the other 5 SNM types.
